# *Schistosoma mansoni* Mass Drug Administration Regimens and Their Effect on Morbidity among Schoolchildren over a 5-Year Period—Kenya, 2010–2015

**DOI:** 10.4269/ajtmh.18-0067

**Published:** 2018-06-11

**Authors:** Anita D. Sircar, Pauline N. M. Mwinzi, Isaac O. Onkanga, Ryan E. Wiegand, Susan P. Montgomery, W. Evan Secor

**Affiliations:** 1 Division of Parasitic Diseases and Malaria, Centers for Disease Control and Prevention, Atlanta, Georgia;; 2 Center for Global Health Research, Kenya Medical Research Institute, Kisumu, Kenya

## Abstract

Schistosomiasis control programs are designed to reduce morbidity by providing mass drug administration (MDA) of praziquantel to at-risk populations. We compared morbidity markers between two cohorts of Kenyan schoolchildren that initially had high prevalence of *Schistosoma mansoni* infections. One cohort (*N* = 416 at year 1) received four rounds of annual MDA in a community-wide treatment (CWT) strategy. The other cohort (*N* = 386 at year 1) received school-based treatment (SBT) every other year over the 4-year period. We measured infection with *S. mansoni* and soil-transmitted helminths (STH) as well as subtle morbidity markers at year 1, year 3, and year 5 and compared cohorts with mixed models after controlling for age and gender. At year 5, neither overall *S. mansoni* prevalence nor the prevalence of high infection–intensity *S. mansoni* infection was significantly reduced compared with baseline in either the CWT cohort (*N* = 277 remaining) or the SBT cohort (*N* = 235 remaining). Nevertheless, by year 5, children in both cohorts demonstrated significant decreases in wasting, ultrasound-detected organomegaly, and STH infection along with significantly improved pediatric quality-of-life scores compared with year 1. Stunting did not change over time, but children who were *S. mansoni* egg–positive at year 5 had significantly more stunting than children without schistosomiasis. The only significant difference between arms at year 5 was a lower prevalence of STH infections in the CWT group.

## INTRODUCTION

*Schistosoma mansoni* infection occurs when skin is exposed to fresh water sources contaminated with the cercarial stage of the parasite.^1^ Pathology of schistosomiasis occurs when deposited schistosome eggs induce an immune-mediated, granulomatous response causing local and systemic inflammation. *Schistosoma mansoni* infection can cause pathology ranging from anemia, growth stunting, and wasting to changes in the liver and spleen, including periportal fibrosis and portal hypertension. Although schistosomiasis is usually not fatal, the cumulative effect of these so-called “subtle morbidities” can impact the quality of life of infected individuals and, as with other neglected tropical diseases, is thought to perpetuate poor economic conditions and delayed cognitive development.^2,3^

The highest prevalence and intensity of schistosome infections usually occur in school-aged children.^4^ In endemic areas, the first infection with schistosomes can be acquired at a very young age.^5^ More than 90% of schistosome infections occur in sub-Saharan Africa,^6^ resulting in at least 3.3 million disability-adjusted life years because of associated morbidities.^7^ In Kenya, approximately six million people have schistosomiasis and an additional 15 million are at high risk of infection.^8^ *Schistosoma mansoni* infection is particularly prevalent in persons living around Lake Victoria and can be hyperendemic in the communities along the shores. We and others have shown that up to 33% of 1-year olds and more than 90% of children > 10 years of age can be infected,^5,9–11^ making this an area in need of schistosomiasis control.

The Schistosomiasis Consortium for Operational Research and Evaluation (SCORE) was established with the overall goal of providing evidence to programs about mass drug administration (MDA) strategies for the control of schistosome infections. Several of the SCORE projects are 5-year longitudinal intervention trials designed to assess different combinations and frequencies of school-based and community-wide MDA treatment strategies.^6^ In addition, SCORE developed nested cohort study protocols to compare the impact of different MDA delivery regimens on selected markers of morbidity.^12^ Markers of morbidity measured included prevalence of stunting (height-for-age z < −2 standard deviation [SD]), prevalence of wasting (body mass index [BMI] for age z < −2 SD), mild–severe anemia (< 5.2–11.2 g/dL), liver abnormalities on ultrasound, maximal oxygen uptake (measured via a 20-m shuttle run), and mean quality-of-life scores. Here, we describe the results of one of these studies evaluating morbidity in cohorts of children living in villages near Lake Victoria after 4 years of intervention compared with baseline. In addition to comparing schistosomiasis control strategies, we hoped to identify measures that could be used to evaluate effectiveness of programs for schistosomiasis morbidity control.

## METHODS

### Study area and population.

**Figure 1.**
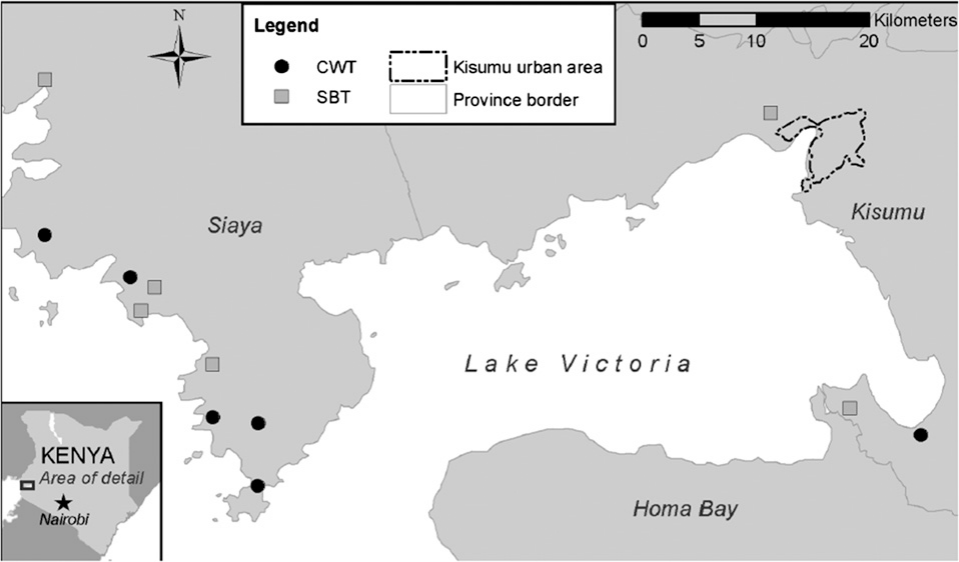
Locations of villages included in cohort study. CWT = community-wide treatment; SBT = school-based treatment.

Selection of the villages included in this study has been described previously.^13^ In brief, 12 communities in Siaya and Kisumu counties (formerly part of Nyanza Province) with ≥ 25% *S. mansoni* infection prevalence among 9- to 12-year-old children were included (Figure 1): six received community-wide treatment (CWT) every year for 4 years and six received school-based treatment (SBT) every other year. Community-wide treatment was delivered house to house by community drug distributors and SBT was delivered in primary schools by health teachers. Children were given a single dose of praziquantel (40 mg/kg) and a single dose of albendazole (400 mg) during treatment years. Children who had evidence of malaria on blood smears were treated with artemether/lumefantrine.

### Ethics statement and eligibility criteria.

Parents and children were asked to provide signed informed consent and assent to participate in the study. Only children who assented and had parental or guardian permission to participate were eligible for inclusion. Children with physical disabilities that compromised their full participation in the study (e.g., the shuttle run) were excluded from the study.

Ethical clearance was obtained from the Departmental and Institutional Scientific Steering Committees of Kenya Medical Research Institute (KEMRI) in Kisumu and Nairobi followed by the National KEMRI Ethical Clearance Committee. Institutional Review Boards of the Centers of Disease Control and Prevention and the University of Georgia also reviewed and approved the study.

### Stool examination.

Each child was asked to provide fresh stool samples on three consecutive days. Samples were processed and examined using the Kato–Katz technique for parasite egg detection at the KEMRI laboratories in Kisumu, Kenya. Two slides per stool sample were examined for a total of six slides per child. The presence of *S. mansoni*, *Ascaris lumbricoides*, *Trichuris trichiura*, and hookworm eggs was recorded. Only egg counts for *S. mansoni* were quantified. The arithmetic mean of egg counts was calculated from the total slides per child and expressed as eggs per gram (epg). Intensity levels were divided into light (1–99 epg), moderate (100–399 epg), and heavy (≥ 400 epg) per WHO guidelines.^14^

### Blood collection and processing.

A 5-mL venous blood sample was collected from each enrolled individual. Hemoglobin concentration was measured in g/dL using a hemoglobinometer (HemoCue AB, Angelholm, Sweden). Anemia was categorized by country-specific cutoffs adjusted for age and altitude with the following values: normal (> 11.2 g/dL), mild (> 8.2–1.2 g/dL), moderate (5.2–8.2 g/dL), and severe (< 5.2 g/dL).^15^ *Plasmodium* spp. parasitemia was assessed via peripheral blood smears examined by trained microscopists.

### Anthropometric measurements and fitness testing.

A stadiometer was used to measure height. Weight was measured with a scale that was calibrated regularly and results were recorded in kilograms. Two readings for height and weight were taken for each child and the mean of those results was calculated. Wasting was defined as BMI-for-age z-score of < −2 SD, and stunting was defined as height-for-age z-score of < −2 SD. Data were entered into the WHO Anthro (version 3.2.2, 2011 for Personal Computers)^16^ software to calculate z-scores. Exercise tolerance was tested using a multistage 20-m shuttle run as a measure of maximal aerobic capacity by correlating the level achieved on running to a maximal oxygen uptake or VO_2_ max. Children were asked to run 20 m at increasing speeds until they were no longer able to continue at the rate set by the recording and then they were asked to stop. This final level achieved correlated with a VO_2_ max measured in mL/kg/minute.^17,18^

### Ultrasonographic evaluations.

An SSD-500 portable ultrasound machine (Aloka, Tokyo, Japan) was used to assess participants for hepatosplenic and portosystemic morbidity according to the Niamey protocol.^19^ Liver texture patterns were graded from A-F. Patterns C-F were considered evidence of schistosomiasis-related fibrosis. Hepatomegaly, splenomegaly, portal branch thickening, and increased portal vein diameter were defined as values two SDs above a reference Senegalese population adjusted for age and height.

### Quality-of-life evaluations.

Quality-of-life evaluations were performed using the Pediatric Quality of Life Inventory^™^ (PedsQL, Lyon, France) for children. Children were individually asked questions in four categories of functioning: physical, emotional, social, and school. The answers were aggregated to create a QL score on a 0–100 continuous scale. Mean scores with 95% confidence limits (CLs) were calculated and compared over the course of the study and between treatment arms.

### Data management and analysis.

Data collected in the field were recorded on smartphones and then uploaded to a central database using a data collection app, EpiCollect (Imperial College, London, England).^20^ Laboratory testing results were recorded on paper forms and entered by a secure web-based portal into the same central database. Data were analyzed using SAS version 9.3 (SAS Institute Inc., Cary, NC). All tests and CLs used the 5% level of significance. Differences in categorical variables were evaluated using the Wald F statistic,^21^ whereas continuous outcomes were evaluated with Taylor series linearization.^22^

## RESULTS

### Study participant characteristics at year 1.

A total of 802 children were enrolled at year 1, 416 in the CWT cohort, and 386 in the SBT cohort. Age and gender ratios were similar between groups (Table 1). The CWT cohort had a higher mean *S. mansoni* prevalence (73.5%) than the SBT cohort (56.2%), but this difference was not statistically significant (Table 2). Other than the difference in wasting prevalence, for which the *P* value was close to the level of significance (*P* = 0.053), there were no significant differences between cohorts in the morbidity markers that we measured at year 1 (Table 2).

**Table 1:** Year 1, 3, and 5 participant characteristics and loss to follow-up by cohort

	Year 1		Year 3		Year 5		Baseline characteristics of children lost to follow-up at year 5	
	CWT	SWT	CWT	SWT	CWT	SWT	CWT	SWT
Number in cohort	416	386	330	311	277	235	130	151
% Male (*n*)	47% (196)	50% (196)	48% (160)	48% (150)	48% (133)	54% (128)	46% (65)	49% (85)
Age in years (SD)	7.5 (0.04)	7.6 (0.02)	9.5 (0.05)	9.8 (0.05)	11.6 (0.05)	11.7 (0.05)	7.5 (0.07)	7.6 (0.04)

CWT = community-wide treatment; SD = standard deviation; SWT = school-based treatment.

**Table 2:** Infection levels and morbidity markers of participants in the community-wide and school-based treatment cohorts at year 1

	CWT		SBT		
Infection	*n*	Percent/value (95% CL)*	*n*	Percent/value (95% CL)*	*P* value
*Schistosoma mansoni* prevalence	407	73.5% (53.9, 93.1)	372	56.2% (26.5, 85.9)	0.33
*S. mansoni* high intensity (≥ 400 eggs per gram)	407	11.8% (2.2, 21.4)	372	9.9% (0.0, 22.5)	0.80
*S. mansoni* mean egg per gram	407	153 (40, 266.7)	372	113 (0, 254.2)	0.56
Malaria prevalence	400	6.3% (3.8, 8.7)	349	9.2% (6.2, 12.1)	0.13
Soil-transmitted helminths (STH) prevalence	276	15.9% (11.0, 20.9)	273	31.5% (12.0, 51.0)	0.13
Morbidity marker					
Prevalence of stunting (height-for-age z < −2 SD)	416	10.8% (3.7, 18.0)	386	7.0% (3.9, 10.1)	0.30
Prevalence of wasting (BMI-for-age z < −2 SD)	416	32.5% (27.3, 37.6)	386	20.5% (11.1, 29.8)	0.053
Mean hemoglobin (g/dL)	400	11.8 g/dL (11.0, 12.7)	348	11.9 g/dL (11.2, 12.5)	0.94
Mild-severe anemia (< 5.2–11.2 g/dL)	400	20.5% (13.7, 27.3)	348	19.3% (13.2, 25.3)	0.77
Normal liver (liver pattern A)	388	72.2% (62.1, 82.3)	348	81.0% (74.0, 88.1)	0.14
Mean VO_2_ (mL/kg/minute)	395	47.7 (47.1, 48.3)	336	47.3 (46.9, 47.6)	0.15
Mean quality-of-life score (PedsQL total)	399	85.9 (84.5, 87.4)	355	85.8 (83.9, 87.7)	0.86

BMI = body mass index; CL = confidence limit; CWT = community-wide treatment; *n* = number of participants; PedsQL = Pediatric Quality of Life Inventory; SBT = school-based treatment; SD = standard deviation; STH = soil-transmitted helminths.

* Data represent the mean and 95% CL of the six villages in each cohort.

### Study participant characteristics at year 5.

At year 5, 277 children (66.6%) remained in the CWT cohort and 235 (60.1%) remained in the SBT cohort; loss to follow-up was primarily attributed to family relocations.^23^ Gender ratios and mean age remained similar between cohorts throughout the study (Table 1).

### Changes in helminth infections and anthropometry over time.

**Figure 2.**
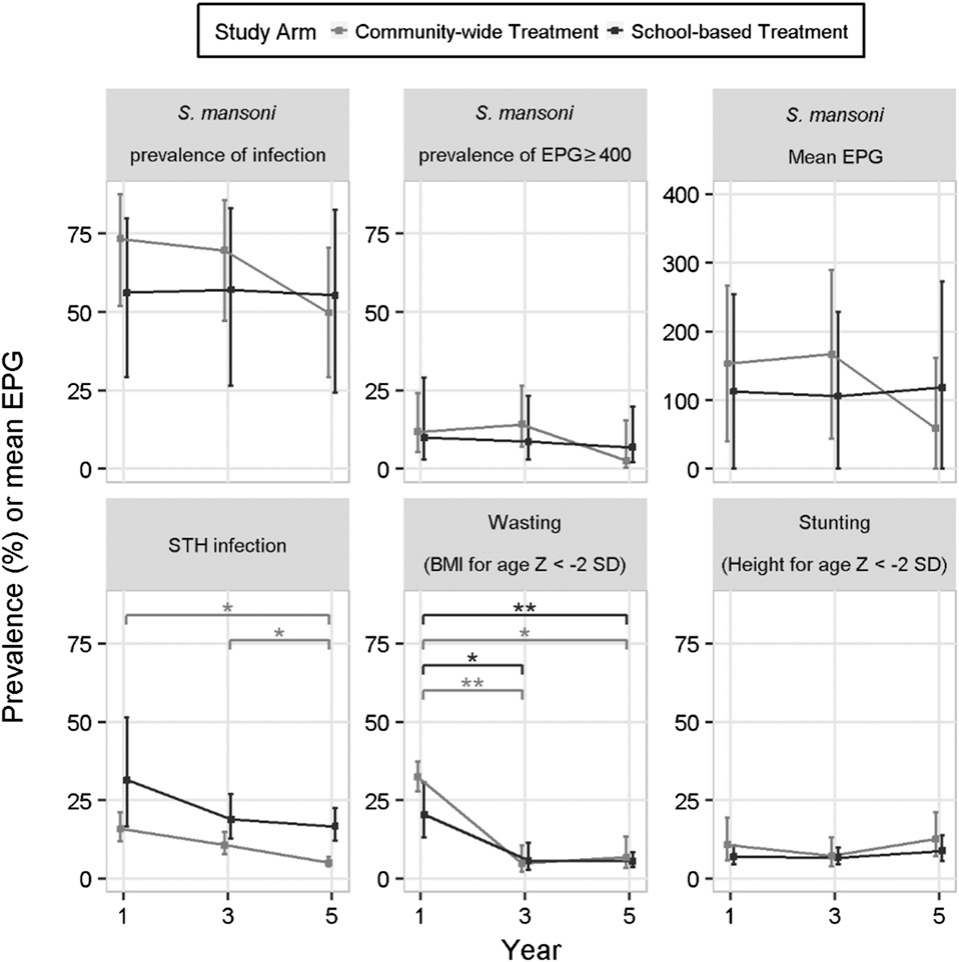
Prevalence of *Schistosoma mansoni* infection and high-intensity infections, soil-transmitted helminth (STH) infection, wasting, and stunting in participants in year 1, 3, and 5 in the community-wide treatment and school-based treatment arms. EPG = eggs per gram; SD = standard deviation (* indicates comparison possessed *P* < 0.05 and ** indicates that comparison had *P* < 0.01).

Neither the CWT nor SBT cohorts had a significant decrease in *S. mansoni* infection prevalence from year 1 to year 5. Similarly, the intensity of infection, whether measured by prevalence of high-intensity (≥ 400 epg) infections or average egg count did not change significantly in either cohort over the course of the study (Figure 2). There were no statistically significant differences in either infection prevalence or intensity between cohorts at years 3 or 5.

Overall prevalence of soil-transmitted helminths (STH) infection in the study was 23.7% in year 1, 14.6% in year 3, and 10.0% in year 5. Soil-transmitted helminths prevalence decreased significantly in the CWT cohort from year 1 to year 5 (*P* = 0.03, Figure 2) and year 3 to year 5 (*P* = 0.047). Soil-transmitted helminths infection levels in the SBT cohort demonstrated a similar pattern, but no significant reductions of prevalence were found.

At enrollment, 26.7% of the children enrolled in the study demonstrated low weight for height, or wasting. Prevalence of wasting decreased over time with statistically significant reductions seen by year 3 for both the CWT and SBT cohorts (*P* = 0.007 and *P* = 0.02, respectively). By contrast, the average prevalence of children demonstrating a reduced growth rate or stunting was much lower at year 1 than the prevalence of wasting and did not change significantly over time for either cohort. However, at year 5, children who were egg positive for *S. mansoni* were more likely to be stunted than children who were egg negative (17.0% versus 5.3%; *P* < 0.001).

### Ultrasonography.

**Figure 3.**
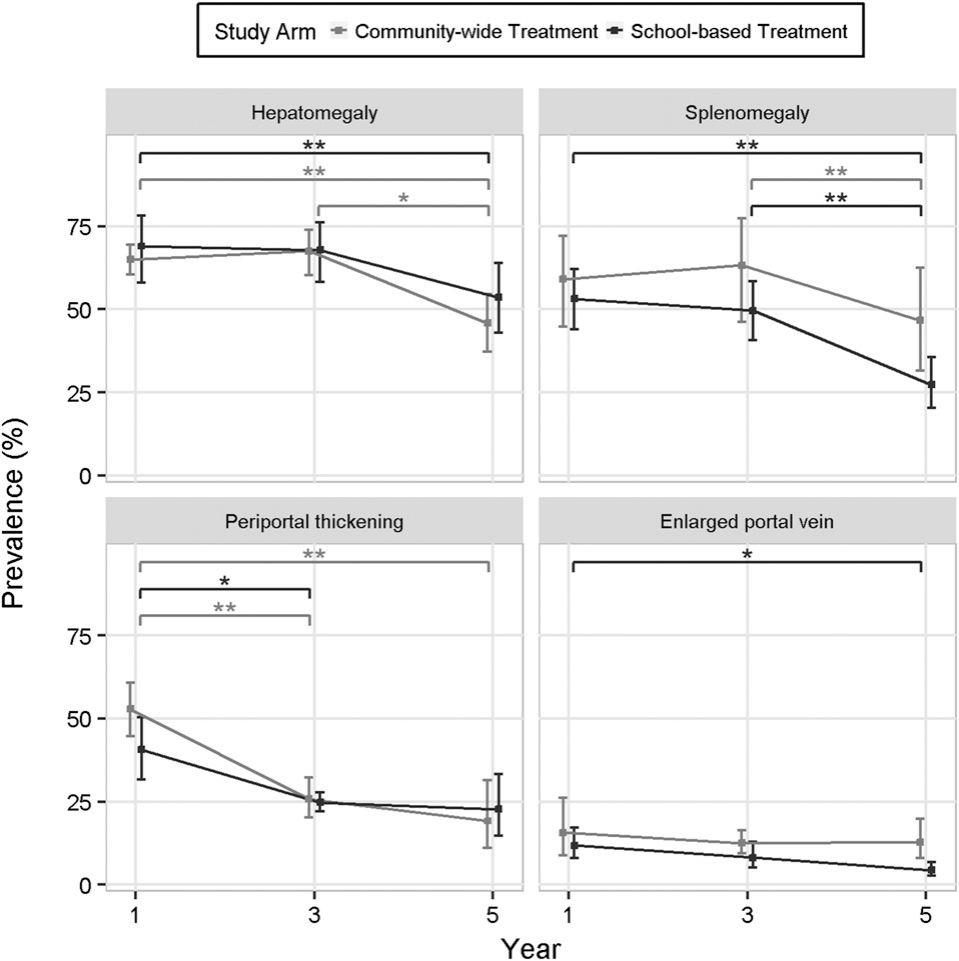
Prevalence of hepatomegaly, splenomegaly, periportal thickening, and enlarged portal vein as demonstrated on ultrasonography in participants in year 1, 3, and 5 in the community-wide treatment and school-based treatment arms (* indicates comparison possessed *P* < 0.05 and ** indicates that comparison had *P* < 0.01).

At baseline, more than 66.9% of all children enrolled in the study had an enlarged liver, 56.3% had an enlarged spleen, 47.1% demonstrated periportal branch thickening, and 13.8% had an enlarged portal vein diameter. Prevalence of hepatomegaly, splenomegaly, and enlarged portal vein diameter did not significantly change between years 1 and 3 of the study (Figure 3). By year 5, however, hepatomegaly was significantly reduced for both cohorts (*P* < 0.008). There were also reductions in spleen size and portal vein diameter, but these were only statistically significant for the SBT cohort (*P* < 0.02 for both measures). Periportal branch thickening decreased significantly by year 3 for both cohorts (*P* < 0.04) and remained low but was only further reduced at year 5 in the CWT arm, not the SBT arm (*P* = 0.007, Figure 3). There were only three study participants who demonstrated schistosomiasis-associated fibrosis (liver pattern C or greater) at year 1, four at year 3, and one at year 5.

### Pediatric quality-of-life scores.

**Figure 4.**
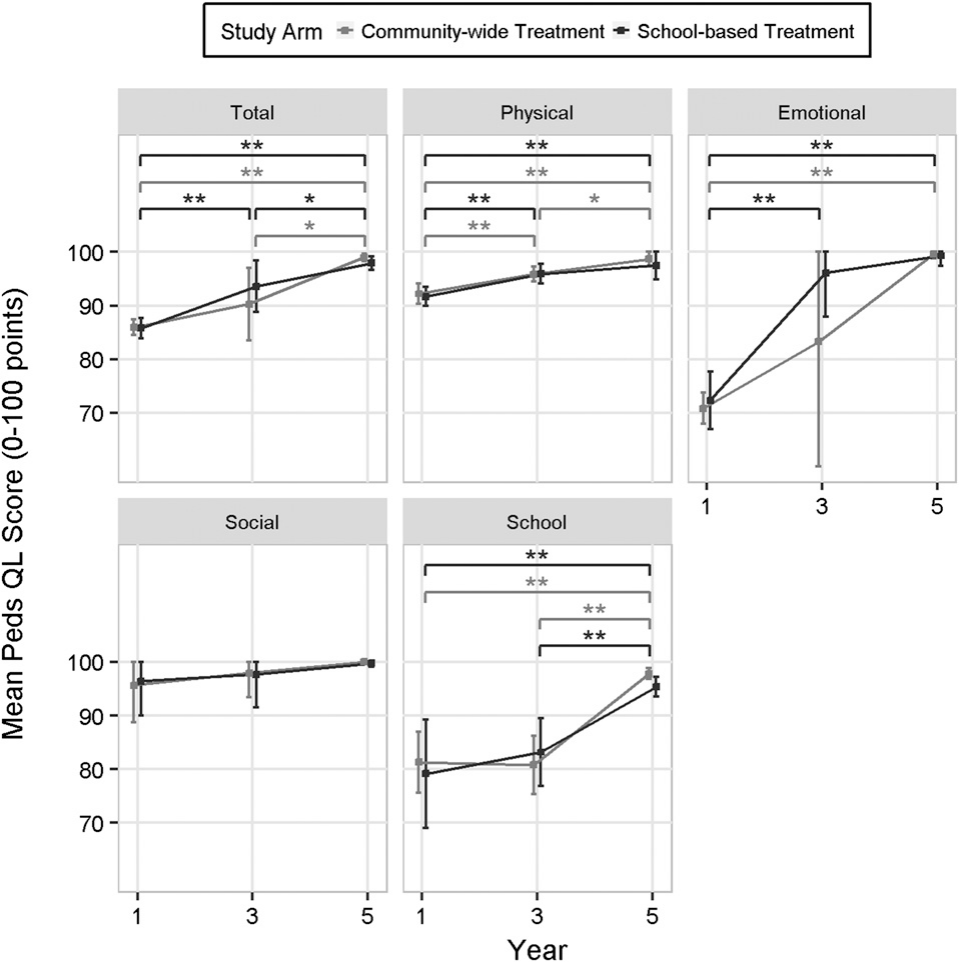
Pediatric quality-of-life (PedsQL) scores from 0 to 100 in participants in year 1, 3, and 5 in the community-wide treatment and school-based treatment arms in the four categories of physical, emotional, social, and school and total score (* indicates comparison possessed *P* < 0.05 and ** indicates that comparison had *P* < 0.01).

Between years 1 and 5 of the study, the total PedsQL score, as well as the scores for the physical, emotional, and school domains increased significantly for both cohorts (*P* < 0.001, Figure 4). Significant improvement for the CWT physical domain (*P* = 0.009) and for the SBT total (*P* = 0.001), physical (*P* = 0.009), and emotional (*P* = 0.001) domains was evident by year 3. There were differences in scores for the school domain in each arm by year 5 (*P* < 0.002). The only domain that did not change over time for either cohort was the social domain; however, at year 1, both cohorts scored > 95% for this domain, leaving little room for improvement.

### Malaria, anemia, and physical fitness.

**Figure 5.**
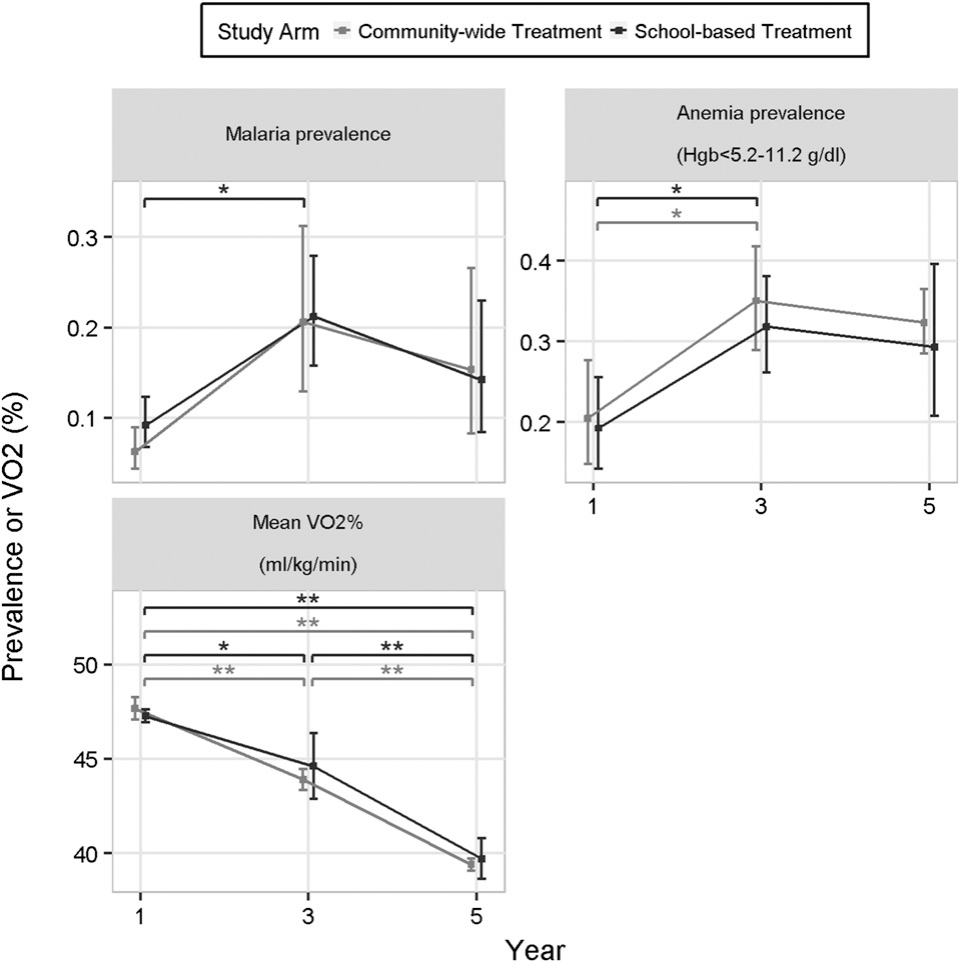
The relationship between malaria infection, anemia, and physical fitness participants in year 1, 3, and 5 in the community-wide treatment and school-based treatment arms. Hgb = hemoglobin, VO_2_ = volume of oxygen consumption (* indicates comparison possessed *P* < 0.05 and ** indicates that comparison had *P* < 0.01).

In addition to being highly endemic for schistosomiasis, the study area also has a high prevalence of malaria (Figure 5). From year 1 to year 3, malaria prevalence among study participants significantly increased from 7.7% to 20.9% in the SBT cohort (*P* = 0.013). At year 5, overall malaria prevalence was 14.8%. Anemia also significantly increased from year 1 to year 3 in both cohorts (*P* < 0.03) and remained elevated at year 5 (Figure 5). By contrast, VO_2_ max, as measured by exercise tolerance, significantly reduced from year 1 to year 3 in both cohorts (*P* < 0.02) and was further reduced at year 5 (*P* < 0.0001, Figure 5).

## DISCUSSION

Although community-wide and school-based MDA regimens did not significantly decrease overall prevalence and intensity of *S. mansoni* infections over time, significant decreases in morbidity as measured by a lower prevalence of wasting, hepatomegaly, portal branch thickening, portal vein diameter, and splenomegaly were observed. Mass drug administration treatment regimens with albendazole for the treatment of STH showed a significant decrease in STH by year 5 in both cohorts. Improvement in morbidity markers such as wasting and PedsQL could partially be the result of decreased STH burden as well; however, it is unlikely that improvements in morbidity documented by ultrasound could be attributed to reduced STH prevalence. The association between schistosomiasis and liver pattern B remains unclear. Previously, we have observed a relationship between pattern B and malaria infection, but not schistosomiasis, in these cohorts.^13^

Stunting did not significantly change over time but at year 5, the prevalence of stunting was significantly higher in children who were positive for *S. mansoni* eggs. Similar findings were extracted in a meta-analysis performed by Andrade et al.^24^ where reduction in egg quantity significantly correlated with a decrease in morbidity. Despite praziquantel therapy not being fully curative, a greater egg count reduction ratio correlated with a greater reduction in odds of most morbidities in their analyses. Olveda et al.^25^ also demonstrated that clinical outcomes of liver fibrosis due to schistosomiasis could be reversed over time with praziquantel treatment. Botelho et al.^26^ showed that schistosome infections can lead to significant, yet largely unacknowledged morbidities such as stunting and wasting, further emphasizing the need for treatment and control measures for schistosomiasis.

Study participants also demonstrated significant improvement using a pediatric quality-of-life inventory for total scores in three of the four specific categories: physical, emotional, and school. Among children in sub-Saharan Africa, Terer et al.^27^ observed significantly lower total PedsQL scores in villages with high schistosomiasis prevalence and those with lower socioeconomic levels. Furthermore, schistosome infections were associated with a 2–4% reduction in total PedsQL scores in their study.

These data suggest that evaluation of morbidity in schistosomiasis control programs could be performed using relatively easy measures of anthropometry and quality-of-life surveys. The limitation of using these measures is that they are not specific for schistosomiasis, and other infections or conditions in developing countries could limit their utility for determining schistosomiasis-specific morbidity. Ultrasonography is more specific but is challenging to conduct in a program setting as expensive equipment, trained personnel, and more time are required to perform it correctly. Our data also indicate that specific organ measurements are more useful in this age group rather than evaluation of overall image patterns. Schistosomiasis-associated fibrosis usually does not develop until infected individuals are older and have had a longer duration of infection. There are new cell phone–based ultrasound devices that could make organomegaly assessments more accessible and would not require onsite presence of a skilled radiologist as images could be transmitted for interpretation.^28^ Whether this technology can be incorporated into morbidity assessments in schistosomiasis control programs will require additional operational research.

By contrast to anthropometry, quality-of-life and ultrasound assessments, anemia, and physical fitness (VO_2_ max) suggested significantly worse health outcomes over the duration of the study, although it is possible that these measures were confounded by the increased prevalence of malaria after year 1. Our data are consistent with Bustinduy et al. who found that decreased aerobic capacity in under-resourced areas was likely due to anemia, stunting, and wasting from chronic parasitic infections.^3^ Our data also suggest that in areas that are endemic for malaria, anemia and exercise tolerance are less reliable measures for schistosomiasis morbidity.

We were unable to detect important differences in markers of morbidity when comparing four rounds of CWT with two rounds of SBT at year 5. The simple explanation may be that biennial treatment provided at the school level is just as effective at reducing schistosomiasis-associated morbidity as annual treatment at the community level when measured in schoolchildren. However, there were several limitations to the study that may have hindered our ability to detect differences between these two cohorts at year 5. One possible explanation for the lack of significant differences in morbidity markers between cohorts by the end of the study is the large variability in *S. mansoni* prevalence and intensity of infection found between the chosen communities at the beginning of the study making it difficult to establish a comparable baseline. Furthermore, the power to detect differences was reduced given that the unit of measurement was the village and each treatment cohort consisted of only six villages. A migratory study population that led to considerable loss to follow-up (33% by year 5 in the CWT cohort and 39% in the SBT cohort) further contributed to the low statistical powering. Had the study been designed with “matched villages,” that is, match a village with ∼25% prevalence in the CWT cohort with a village in the SBT cohort with ∼25% prevalence, it may have been possible to detect a difference between treatment regimens. However, in the parent study that consisted of 25 villages each receiving annual CWT or biennial SBT from which the six villages in each cohort were a subset, there were no statistically significant differences in the final prevalence or intensities of infection between these two treatment regimens, although there were highly significant reductions in prevalence and intensity of schistosome infections over time for each treatment strategy (manuscript in preparation). The results of this study were not sufficiently powered to make any definitive recommendations for either regimen being preferable over the other. Future studies with different sampling frames, increased sample size, and better balanced village-level prevalence of *S. mansoni* infection at baseline are needed to provide definitive program guidance.

The absence of a significant lowering of *S. mansoni* prevalence and intensity of infection over time in either cohort may reflect the small numbers of villages that were included in these cohorts and the large variability in *S. mansoni* prevalence and intensity at baseline. However, treatment was still beneficial to the individual as evidenced by reduced schistosomiasis-associated morbidity over the course of the study. If SBT given two times over 4 years provides the same benefits in reduced morbidity as giving annual CWT, the lower cost SBT regimen may be the more cost-effective approach. Our results offer information that may be used to develop more conclusive studies and suggest that in areas co-endemic for malaria, anthropometry, quality-of-life questionnaires, and perhaps ultrasound would be useful measures of morbidity to evaluate the public health impact of schistosomiasis control programs. Further research on the association between infection levels during the course of MDA and schistosomiasis-associated morbidity is needed to better define program targets and improve the guidelines for schistosomiasis control.
